# Phenomenological examinations of delirium in advanced cancer patients: exploratory structural equation modelling and latent profile analysis

**DOI:** 10.1186/s12904-020-00668-0

**Published:** 2020-10-19

**Authors:** Eun-Jung Shim, Hyeju Ha, Won-Hyoung Kim, Moon-Hee Lee, Jisun Park, Kwang-Min Lee, Kyung-Lak Son, Chan-Woo Yeom, Bong-Jin Hahm

**Affiliations:** 1 Department of Psychology, Pusan National University, Busan, Republic of Korea; 2 Department of Psychiatry, Inha University Hospital, Incheon, Republic of Korea; 3 Department of Hematology-Oncology, Inha University Hospital, Incheon, Republic of Korea; 4 Mind Lab The Place, Seoul, Republic of Korea; 5 Department of Psychiatry, Dongguk University Ilsan Hospital, Goyang, Republic of Korea; 6 Department of Psychiatry, National Rehabilitation Center, Seoul, Republic of Korea; 7 Department of Neuropsychiatry, Seoul National University Hospital, Seoul, Republic of Korea; 8 Department of Psychiatry and Behavioral Sciences, Seould National University College of Medicine, Seoul, Republic of Korea

**Keywords:** Cancer, Delirium, Exploratory structural equation modelling, Latent profile analysis, Palliative care

## Abstract

**Background:**

This study examined phenomenological manifestations of delirium in advanced cancer patients by examining the factor structure of the Delirium Rating Scale-Revised-98 (DRS-R-98) and profiles of delirium symptoms.

**Methods:**

Ninety-three patients with advanced cancer admitted to inpatient palliative care units in South Korea were examined by psychiatrists using the DRS-R-98 and the Confusion Assessment Method (CAM). The factor structure of the DRS-R-98 was examined by exploratory structural equation modelling analysis (ESEM) and profiles of delirium were examined by latent profile analysis (LPA).

**Results:**

CAM-defined delirium was present in 66.6% (*n* = 62) of patients. Results from the ESEM analysis confirmed applicability of the core and noncore symptom factors of the DRS-R-98 to advanced cancer patients. LPA identified three distinct profiles of delirium characterizing the overall severity of delirium and its core and noncore symptoms. Class 1 (*n* = 55, 59.1%) showed low levels of all delirium symptoms. Class 2 (*n* = 17, 18.3%) showed high levels of core symptoms only, whereas Class 3 (*n* = 21, 22.6%) showed high levels of both core and noncore symptoms except motor retardation.

**Conclusions:**

Clinical care for delirium in advanced cancer patients may benefit from consideration of the core and noncore symptom factor structure and the three distinct phenomenological profiles of delirium observed in the present study.

## Background

Delirium in palliative care, especially in an inpatient palliative care setting, is a more serious and prevalent concern. A recent systematic review and meta-analysis indicated that up to 74% of patients experience delirium in inpatient palliative care units and its prevalence goes up to 88% approaching death. Pooled prevalence estimates indicated that one third of patients were diagnosed with delirium at the time of admission to inpatient palliative care [1]. This high prevalence reflects that patients in palliative care settings are more frail, with poor performance status [2]. It was suggested that delirium-induced disinhibition may result in the overexpression and worsening of physical and psychological symptoms [3]. It also affects survival length [2], and it distresses the family members of terminal cancer patients [4].

An examination of the phenomenology of delirium in advanced cancer patients with a valid assessment tool is the first step to relieve such a burden. To this end, several assessment tools for delirium have been applied in palliative care settings. The Confusion Assessment Method [5] and the Memorial Delirium Assessment Scale (MDAS) [6] are commonly used and serve as the recommended assessment tools in palliative care [7, 8]. The Delirium Rating Scale-Revised-98 (DRS-R-98) [9], which was developed based on theory and clinical experiences [10], is another important assessment tool, and it is considered to be the most detailed phenomenological tool available, allowing both the diagnosis of delirium and detailed assessments of the severity of its symptoms [11].

The applicability of the DRS-R-98 in palliative care settings was partly demonstrated by its good overall agreement with the MDAS [12]. Yet, as a previous review indicated, there have been no validity examinations for the DRS-R-98 in the palliative care setting [13], and in particular, limited studies examining its construct validity with advanced cancer patients.

On the other hand, the validity of the DRS-R-98 has been examined in other patient populations and settings. In particular, the factor structure of the DRS-R-98 has been the subject of previous examinations with some varying results regarding the number of factors, mostly indicating either two [14, 15] or three factors [16, 17]. For instance, Franco et al. [14] identified the “Cognition, and Psychosis/Agitation factors” in a study with 161 surgical patients, and Jain et al. [15] proposed the cognitive, and behavioral factors in their study with 86 referred patients with delirium. On the other hand, Grover et al. [16] and Matoo et al. [17], in their studies with patients with confirmed diagnoses of delirium, proposed three factors for the DRS-R-98: “global cognitive,” “sleep and motor symptoms,” and the “thought and language” factors for the first; and “cognition”; “circadian & psychosis,” and “higher order thinking” factors for the latter. A factor structure similar to that suggested by Matoo et al. [17] was also observed in a study by Franco et al. [18]. Using a pooled international dataset of 592 patients, they proposed that the core symptoms of delirium as assessed by the DRS-R-98 is consisted of three core domains: circadian, cognitive, and higher level thinking. All these findings appear to be somewhat synthesized into the two factor structure of the DRS-R-98, i.e., the core and noncore symptom factors proposed by the latest examination with the data from 859 adult patients in a multisite pooled international delirium database by Thurber et al. [10]. Core symptoms such as disturbances in attention and other cognitive functions represent common and consistent features of delirium, whereas noncore symptoms represent less common and more variable features of delirium such perceptual disturbances or lability of affect [18].

In view of this, the present study aimed to examine whether the core and noncore factors proposed by Thurber et al. [10] is applicable to advanced cancer patients in an attempt to explore the phenomenology of delirium as observed in advanced cancer patients [15]. In so doing, the present study applied the exploratory structural equation modeling (ESEM) method, which is an integration of confirmatory factor analysis (CFA), exploratory factor analysis, and structural equation modeling [19]. It has been suggested that as clinical symptoms tend to be correlated, the CFA requirement of the restriction of zero cross-loadings might be restrictive, leading to potentially biased estimates [19, 20]. When non-zero cross-loadings are specified as zero as in the CFA, it may lead to over-estimated factor correlations and subsequent distorted structural relations. For this reason, it was considered important to extend structural equation modeling to permit less restrictive measurement models that can be used together with the CFA models, which is ESEM [21]. ESEM is considered to be appropriate in clinical studies in which traditional factor analyses may not be appropriate [19].

In addition, the present study examined profiles of delirium symptoms through latent profile analysis, a person-centered approach that identifies homogenous subgroups of individuals based on the pattern of the means of observed variables, i.e., individual delirium symptoms as assessed by the DRS-R-98 in the present study [22]. Understanding of subgroups of patients who share similar patterns of delirium symptoms may allow for a tailored approach to interventions in advanced cancer patients with delirium.

Therefore, the present study examined phenomenological manifestations of delirium by examining the factor structure of the DRS-R-98 and profiles of delirium symptoms in advanced cancer patients receiving palliative care.

## Methods

### Participants and procedures

Patients were consecutively recruited from two inpatient palliative care units in South Korea between August 2018 to July 2019. Attending psychiatrists in these 2 units assessed delirium symptoms of admitted patients using the DRS-R-98 and the Confusion Assessment Method (CAM) upon obtaining informed consent. A total of 93 patients were assessed, of which 73 patients were from the palliative care unit of a university hospital, and 20 patients were from the palliative care unit in a provincial medical center. This study was approved by two Institutional Review Boards (IRB No. H-1809-105-974, and IRB No. 2018–07-006).

### Measures

The DRS-R-98 is a composed of 13 items assessing the severity of delirium symptoms and 3 diagnostic items (i.e., temporal onset, fluctuation of symptoms, and physical disorder) [9]. Severity items include the sleep-wake cycle, perceptual disturbances, delusions, lability of affect, language, thought process, motor agitation or retardation, orientation, attention, and short- and long-term memory, and visuospatial ability. Symptoms are rated on a four-point scale (0–3) with a score range of 0 to 39. A severity scores of over 15 is considered to be a case of delirium. The Cronbach’s alpha of the DRS-R-98 total and severity scale were 0.90 and 0.87, respectively, and the interrater reliability (the intraclass coefficient) ranged from 0.98 to 0.99 [9].

The CAM was also administered to define the diagnosis of delirium. The CAM has 9 items, four of which are diagnostic items (i.e., acute onset, inattention, disorganized thinking, and altered level of consciousness) [5]. The inter-rater reliability of the CAM (kappa) was between 0.81 to 1.0 [5].

### Statistical analyses

The two-factor structure (i.e., core and noncore symptoms of delirium) of the DRS-R-98 was examined by the ESEM, and model-fit indices were examined. The criteria to evaluate the goodness-of-fit indices of the model were the following: root mean square error of approximation (RMSEA) ≤ .08, 90% confidence interval (CI) of RMSEA; comparative fit index (CFI) ≥ .95; Tucker-Lewis Index (TLI) ≥ .95; and standardized root mean square residual (SRMR) ≤ .08 [23].

Patterns of delirium symptoms were examined by latent profile analysis (LPA) [22]. Thirteen severity symptoms of the DRS-R-98 were used as indicators. To determine the optimal number of latent classes, three information criterion indices (i.e., Akaike Information Criterion, AIC; Bayesian Information Criterion, BIC; and sample-size-adjusted BIC, SA-BIC) were evaluated with smaller values of the all these indices indicating the better model fit [24]. The classification accuracy was evaluated by the entropy with the larger value indicating a better accuracy [25], and its values above 0.80 were considered adequate [26]. Lastly, the Lo-Mendell-Rubin likelihood ratio test (LMR) [27] and bootstrap likelihood ratio test (BLRT) [24] were examined to determine whether a *k* profile solution fits better than a *k* − 1 profile solution. A significant value *p*-value indicates a fit improvement with the addition of the class [24].

Predictors of the latent class membership were examined using multinomial logistic regression analyses with R3STEP method [28]. Statistical analyses were performed using the IBM SPSS statistical package (version 25.0) and the Mplus software (version 8.3).

Potential differences between the classes in the total and severity scores of the DRS-R-98, and the core and noncore symptoms was examined by the analysis of variance (ANOVA).

## Results

### Participant characteristics and symptoms of delirium

A total of 93 patients with advanced cancer receiving palliative were examined, and their sociodemographic characteristics are shown in supplementary Table 1. The majority of patients were male (52, 57.1%) and married (65, 73.9%). Delirium as diagnosed by the CAM criteria was present in 66.6% (*n* = 62). The delirium and non-delirium groups significantly differed in terms of age (72.9 versus 66.6; *t* = 2.28, *p* < .05) and hospitalization period (9 days versus 2.5 days; *t* = 3.15, *p* < .01).

Means and frequency of individual delirium symptoms, the means of the DRS-R-98 total, and severity scores of the patients are shown in Table 1. The total and severity scores were higher in the delirium group than in the non-delirium group (22.343 vs 5.23 for total scores, *t* = 13.42, *p* < .001; 17.46 vs 2.65 for severity scores, *t* = 12.20, *p* < .001).

**Table 1:** DRS-R-98 items means and frequencies (*N* = 93)

	Total (*N* = 93)		Delirium (*n* = 62)		No Delirium (*n* = 31)	
DRS-R-98-item	Score ≥ 1, %	Mean score (SD)	score ≥ 1, %	Mean score (SD)	score ≥ 1, %	Mean score (SD)
1. Sleep-wake cycle	78.5	1.56 (1.08)	93.5	2.06 (.88)	48.4	.55 (.62)
2. Perceptual disturbances	40.2	.79 (1.14)	60.7	1.20 (1.22)	0.0	.00 (.00)
3. Delusions	22.8	.36 (.74)	34.4	.54 (.85)	0.0	.00 (.00)
4. Lability of affect	35.5	.57 (.88)	50.0	.79 (.93)	6.5	.13 (.56)
5. Language	49.5	.86 (1.06)	74.2	1.29 (1.06)	0.0	.00 (.00)
6. Thought process	62.4	1.04 (1.02)	88.7	1.48 (.90)	9.7	.16 (.58)
7. Motor agitation	47.3	.87 (1.12)	62.9	1.23 (1.19)	16.1	.16 (.37)
8. Motor retardation	40.9	.73 (1.05)	48.4	.97 (1.19)	25.8	.26 (.44)
9. Orientation	64.1	1.33 (1.23)	88.5	1.89 (1.08)	16.1	.23 (.62)
10. Attention	79.6	1.45 (1.08)	100.0	1.98 (.88)	38.7	.39 (.50)
11. Short-term memory	71.7	1.27 (1.07)	90.2	1.70 (.97)	35.5	.42 (.67)
12. Long-term memory	43.5	.70 (.92)	57.4	.93 (.96)	16.1	.23 (.62)
13. Visuospatial ability	54.3	1.02 (1.16)	78.7	1.48 (1.12)	6.5	.13 (.56)
14. Temporal onset	81.7	1.46 (.80)	100.0	1.94 (.31)	45.2	.52 (.63)
15. Fluctuation of symptom	68.8	.71 (.50)	100.0	1.03 (.18)	6.5	.06 (.25)
16. Physical disorder	100.0	2.00 (.00)	100.0	2.00 (.00)	100.0	2.00 (.00)
DRS-R-98 Severity score		12.47 (9.99)		17.46 (8.45)		2.65 (3.06) ^***^
DRS-R-98 Total score		16.63 (10.93)		22.43 (8.53)		5.23 (3.74) ^***^

*Note.* ^***^ *p* < .001

### Factor structure of the DRS-R-98

Examination of the two factor structure (i.e., core and noncore symptoms of delirium) applied to ESEM yielded an adequate fit for the model (*Χ*^2^_(73)_ = 134.8, *p* < .001; *RMSEA* = .095 [.070–.120]; *CFI* = .951; *TLI* = .930; and *SRMR* = .037). Two diagnostic items (i.e., temporal onset and fluctuation of symptoms), and the core symptoms (i.e., sleep wake cycle, language, thought process, motor retardation, orientation, attention, short-term and long-term memory, and visuospatial ability) were loaded on the factor 1, and the noncore symptoms (i.e., perceptual disturbances, delusions, lability of affect, and motor agitation) were loaded on the factor 2 (Table 2). The correlation between factors 1 and 2 was .44.

**Table 2:** Factor loading matrix of the ESEM model of DRS-R-98 (*N* = 93)

DRS-R-98-item	Factor 1	Factor 2
1. Sleep-wake cycle	.69^***^	.29^***^
2. Perceptual disturbances	.18*	.64^***^
3. Delusions	.19*	.50^***^
4. Lability of affect	−.03	.75^***^
5. Language	.87^***^	−.08
6. Thought process	.70^***^	.24^***^
7. Motor agitation	.17*	.69^***^
8. Motor retardation	.69^***^	−.35^***^
9. Orientation	.90^***^	.09
10. Attention	.70^***^	.31^***^
11. Short-term memory	1.00^***^	−.20^**^
12. Long-term memory	.95^***^	−.35^***^
13. Visuospatial ability	.91^***^	−.08
14. Temporal onset of symptom	.59^***^	.21^*^
15. Fluctuation of symptom	.50^***^	.33^***^

*Note*. Factor loading of > .40 are bolded;

* *p* < .05, ** *p* < .01, *** *p* < .001

### Profiles of delirium symptoms

**Fig. 1:**
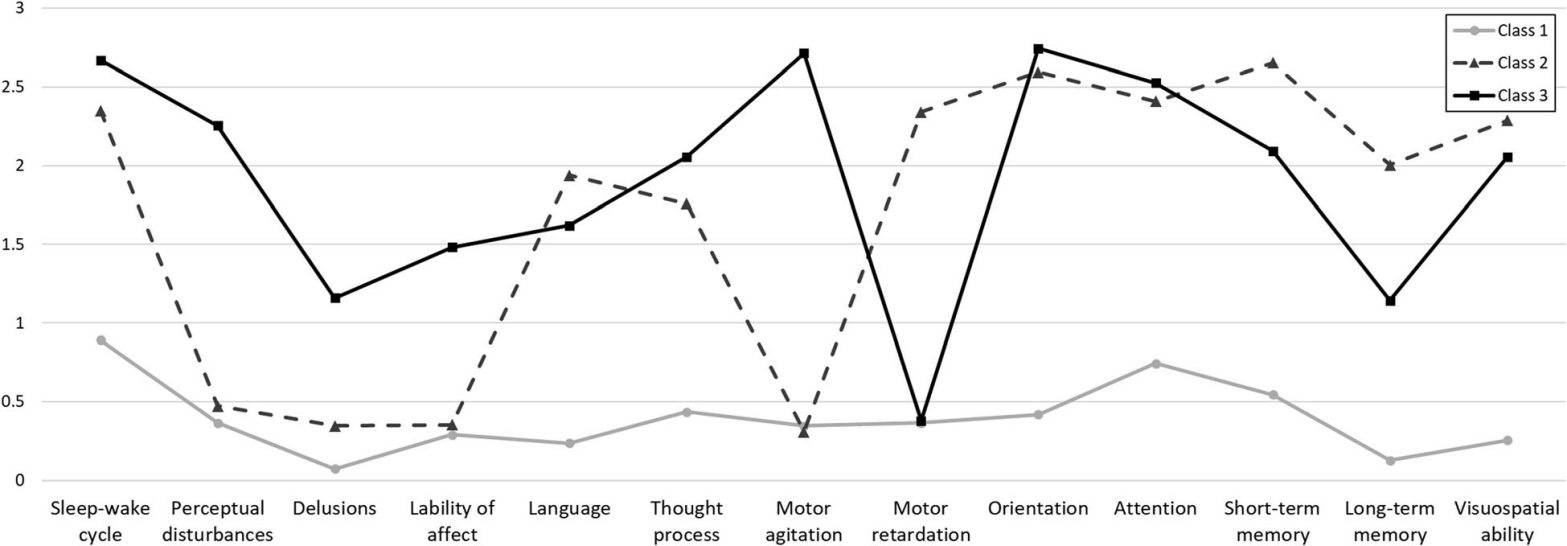
Latent profiles of delirium symptoms

**Fig. 2:**
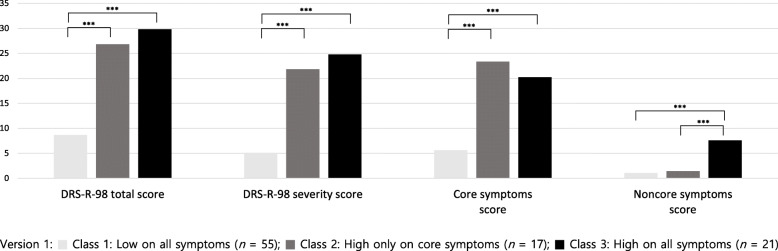
The DRS-R-98 total and severity scores and the core and noncore symptoms among the classes.  Class 1: Low on all symptoms (*n* = 55);  Class 2: High only on core symptoms (*n* = 17);  Class 3: High on all symptoms (*n* = 21)

Results of the latent profile analysis indicated that the models converged up to three classes (Table 3). The three-class solution was deemed to be the best based on an overall examination of model fit indices. Three profiles of delirium symptoms are depicted in Fig. 1 and supplementary Table 2. The classes were characterized by the overall severity of delirium as well as severity of the core and noncore symptoms. Class 1 (*n* = 55, 59.1%) was characterized by low levels of all delirium symptoms. Class 2 (*n* = 17, 18.3%) was characterized by its high levels of core delirium symptoms only, whereas Class 3 (*n* = 21, 22.6%) showed high levels of both core and noncore symptoms except the motor retardation. The ANOVA results indicated that Classes 2 and 3 significantly differed only in the level of noncore symptoms, *F* = 108.41, *p* < .001, *M* = 1.5 (*SD* = 1.8) vs 7.6 (*SD* = 2.3), respectively, but not in core symptoms or total and severity scores (Fig. 2). The percentage of patients with CAM defined delirium was 45.5% (25/55), 94.1% (16/17), and 100% (21/21) for Classes 1, 2 and 3, respectively.

**Table 3:** Fit indices for latent profile analysis models

Model	AIC	BIC	SABIC	LMRLRT (*p*)	BLRT (*p*)	Entropy
1-class	3532.36	3598.20	3516.13			
2-class	2827.82	2929.12	2802.85	.000	.000	1
3-class	2632.72	2769.48	2599.01	.258	.000	.992

*Notes*. *AIC* Akaike information criteria, *BIC* Bayesian information criteria, *SABIC* Sample-size adjusted BIC, *LMRLRT* Lo, Mendell, & Rubin (2001) test, *BLRT* Bootstrapped log-likelihood ratio test

The results of multinomial logistic regression analysis indicated that only the length of hospitalization significantly predicted the class membership (Table 4). Patients with a longer hospitalizations were more likely to be in Class 2 than in Class 3 (*OR* = 1.127; *p* < .05), or more likely to be in Class 2 than in Class 1 (*OR* = .957; *p* < .05).

**Table 4:** Predictors of the class membership

	Class 1 vs. Class 2^a^			Class 1 vs. Class 3^a^			Class 2 vs. Class 3^a^		
Predictors	Coef.	*SE*	*OR*	Coef.	*SE*	*OR*	Coef.	*SE*	*OR*
Age	.013	.033	1.013	−.029	.031	.972	−.041	.043	.960
Sex	−.475	.657	.622	−.032	.758	.968	.443	.931	1.557
Education	.949	1.089	2.583	−.442	.518	.643	−1.391	1.250	.249
Marital status	.106	.687	1.111	.478	.770	1.613	.373	.931	1.452
Religion	.384	.742	1.467	−1.065	.616	.345	−1.448	.915	.235
Hospitalization period (days)	−.044^*^	.019	.957	.075	.057	1.078	.120^*^	.056	1.127

*Notes.* ^a^Reference group; Class 1: low on all symptoms; Class 2: high only on core symptoms; Class 3: high on all symptoms; *Coef*. the estimate (*β*) from the R3STEP multinomial logistic regression; *SE* Standard error of the coefficient, *OR* Odds ratio; Analyses were conducted with the data from 86 patients due to listwise deletion

* *p* < .05

## Discussion

### Summary of findings

The present study examined phenomenological manifestations of delirium symptoms in 93 advanced cancer patient from two inpatient palliative care units in South Korea. Specifically, the core and noncore symptom factor structure of the DRS-R-98 suggested by Thurber et al. [10] was examined. Moreover, patterns of delirium symptoms were examined by latent profile analysis.

The results of the ESEM supported the two previously observed separate but correlated factors of delirium, i.e., the core and noncore symptoms of delirium [10]. A single factor comprised of core symptoms concurs with the previous EFA results including only core symptoms which were the circadian, higher level thinking, and cognitive symptoms [18].

Consistent with the loading pattern previously observed, two diagnostic items and core symptoms were loaded on factor 1 whereas noncore symptoms were loaded on factor 2 [10]. Regarding the loading of the motor agitation on the noncore symptoms, Thurber et al. [10] suggested that while a motor agitation (i.e., hyperactivity) is a component of circadian rhythm, it may also be affected by similar neurophysiological mechanisms (e.g., excess of dopaminergic activity) behind noncore symptoms.

Yet, the magnitude of loadings of specific symptoms in the core symptom factors slightly differed, and memory deficits (i.e., short- and long-term) had high loadings in contrast to the previous findings indicating the highest loading of attention on the core symptoms [10]. Memory deficits are quite common in the phenomenology of delirium with the rates of 88–96% [29]. In fact, the occurrence of short- and long-term memory deficits (88 and 89%, respectively) was the second highest after those of attention and sleep wake cycle disturbances, both of which were 97% in severity according to a previous assessment of 100 delirium cases [30]. Short-term memory impairment was also prevalent as a moderate to severe degree of impairment was observed in 90% of cancer patients with delirium [31]. Attention deficit, an essential diagnostic criterion, was a prominent symptom in Classes 2 and 3, in which almost all patients had the delirium, confirming that it is a cardinal feature of delirium.

Apart from sleep-wake cycle disturbances, the noncore symptom factors in the present study concurs with the “sleep and motor symptom” factor in the previous finding with 151 delirium patients [16] and the “behavioral” factor proposed in a study with 86 referred patients [15].

The core and noncore symptom factors seemed to be reflected in the LPA results. Latent profile analysis with individual symptoms from the DRS-R-98 identified the three distinct classes. These classes were characterized by the overall severity of delirium as well as the severity of the core and noncore symptoms. Class 1 showed low levels of all delirium symptoms. Class 2 showed high levels of core delirium symptoms but not noncore symptoms, whereas the Class 3 showed high levels of both core and noncore symptoms except motor retardation. The ANOVA results confirmed that Classes 2 and 3 significantly differed only in their levels of noncore symptoms. With regard to motor symptoms, Classes 2 and 3 showed opposite patterns. While motor retardation was at its peak in Class 2, motor agitation peaked in Class 3. It may be that these classes reflect hypoactive and hyperactive or mixed subtypes of delirium.

The length of hospitalization was a significant predictor of the class membership, and a longer length of stay was generally associated with the classes having a greater severity of delirium.

### Clinical implications

The DRS-R-98 is known to be developed based on clinical presentations of symptoms in patients with delirium which are not sufficiently reflected in the DSM-IV diagnostic criteria [10]. The current examinations supported previously observed and distinct presentations of the core and noncore symptoms of delirium [10]. Along with the overall severity of delirium, the severity of core and noncore symptoms characterized three latent profiles of delirium, which suggest that it may be helpful to examine not only the total scores but also patterns of core and noncore symptoms.

Adequate assessment and subsequent treatment of core symptoms are important as the core symptoms in orientation, short- and long-term memory, attention, and thought processes prominently characterized persistent delirium in a longitudinal examination of patients with delirium admitted to a palliative care unit [32]. Similarly, the severity of long-term memory during the delirium episode was associated with a longer duration of delirium in a previous longitudinal examination [33].

The evaluation of noncore symptoms serves its purpose in the clinical management of delirium. For instance, noncore symptoms such as perceptual disturbances, lability of affect, and motor agitation were among the symptoms that distinguished both delirium and prodromal delirium from non-delirium in a study of 161 surgical patients [14]. Moreover, the noncore symptoms from the DRS-R-98 differed between patients with a mixed subtype and those with a hypoactive subtype in a study with 321 referred patients to a consultation-liaison psychiatric consultation [34], and in particular, the occurrence and severity of perceptual disturbances and delusions differed between the hyperactive and hypoactive subtypes of delirium in a study with 100 cancer patients [35]. Hypoactive and mixed subtypes of delirium are known to be common in palliative care patients, and one review indicated that hypoactive delirium is the most prevalent subtype of delirium in palliative care [36]. As the hypoactive type is associated with persistent delirium [37], and increased mortality [38], its prompt recognition is important. Still, the misdiagnosis of the hypoactive subtype of delirium as depression or fatigue often occurs in palliative care patients at the terminal stage [7]. These findings suggest that assessment of noncore symptoms may be useful for the early detection of full delirium as well as correct diagnosis of delirium.

Taken together, the current findings suggest the need for tailored management of delirium with advanced cancer patients in the palliative care settings, considering differences in the phenomenological manifestations of delirium.

### Limitations

This study has several limitations. A relatively small sample size may have affected the statistical power of analyses such as ESEM and LPA, as well as the generalizability of the findings. Moreover, although a previous study suggests that the phenomenology of the DRS–R-98 was not different with or without comorbid cognitive impairment in patients with delirium [30], information about comorbidities such as dementia, depression, and other psychiatric or physical conditions, which may have affected phenomenological manifestations of delirium, were unavailable in this study. Moreover, in the absence of information, clinical characteristics (e.g., cancer stage and performance status) associated with class membership as well as the potential impact of identified profiles of delirium on outcomes such as mortality or patient reported outcomes could not be examined. Furthermore, this study did not examine different subtypes of delirium, which may have helped to better understand the classes identified in this study.

## Conclusions

Nonetheless, the present study attempted the phenomenological examination of delirium as experienced in advanced cancer patients, applying analytic methods such as the exploratory structural equation modelling, which may be more suitable for studying clinical conditions such as delirium, and latent profile analysis, a person-centered analytical approach which allowed the identification of three subgroups of patients based on their patterns of individual delirium symptoms.

Clinical care for the highly distressing condition of delirium prevalent in advanced cancer patients may benefit from the consideration of the core and noncore symptom factor structure and the three distinct phenomenological profiles of delirium observed in the present study.

## Supplementary information

**Additional file 1: TableS1.** Participant characteristics (*N* = 93). **Table S2.** Delirium symptom levels in latent profiles

## Data Availability

The datasets used and/or analyzed during the current study are available from the corresponding author on reasonable request.

## Abbreviations

AIC: Akaike Information Criterion,
BIC: Bayesian Information Criterion
BLRT: Bootstrap likelihood ratio test
CAM: Confusion assessment method
CFA: Confirmatory factor analysis
CFI: Comparative fit index
CI: Confidence interval
DRS-R-98: Delirium Rating Scale-Revised-98
EFA: Exploratory factor analysis
ESEM: Exploratory structural equation modeling
LMR: The Lo-Mendell-Rubin likelihood ratio
LPA: Latent profile analysis
RMSEA: Root mean square error of approximation
SA-BIC: S sample-size-adjusted BIC
SRMR: Standardized root mean square residual
TLI: Tucker-Lewis Index
