# Supplementary material for: Phenomenological examinations of delirium in advanced cancer patients: exploratory structural equation modelling and latent profile analysis
Source: BMC Palliat Care. 2020 Oct 19;19:162. doi: 10.1186/s12904-020-00668-0 (PMC7574192; doi:10.1186/s12904-020-00668-0)
Supplement: Supplementary file 1 — Additional file 1: TableS1. Participant characteristics (N = 93). Table S2. Delirium symptom levels in latent profiles [file 12904_2020_668_MOESM1_ESM.docx]

Supplementary Table1. Participant characteristics (*N* = 93)

|  | Total  (*N* = 93) | Delirium^†^  (*n* = 62) | No Delirium^†^  (*n* = 31) |  | |
| --- | --- | --- | --- | --- | --- |
| Variables | *M* (*SD*) or *N* (%) | *M* (*SD*) or *N* (%) | *M* (*SD*) or *N* (%) | *t*/*χ^2^* | |
| Age (*n* = 92) | 70.76 (12.73) | 72.87 (11.82) | 66.61 (13.60) | 2.28^*^ | |
| Sex (*n* = 91) |  |  |  |  | |
| Female | 39 (42.9) | 28 (46.7) | 11 (35.5) | 1.04 | |
| Male | 52 (57.1) | 32 (53.3) | 20 (64.5) |  |  |
| Education (years; *n* = 88) |  |  |  |  | |
| < 6 | 31 (35.2) | 23 (40.4) | 8 (25.8) | 3.40 | |
| 6-12 | 48 (54.5) | 27 (47.4) | 21 (67.7) |  |  |
| > 12 | 9 (10.2) | 7 (12.3) | 2 (6.5) |  |  |
| Marital status (*n* = 88) |  |  |  |  | |
| Not married^‡^ | 23 (26.1) | 14 (24.6) | 9 (29.0) | .21 | |
| Married | 65 (73.9) | 43 (75.4) | 22 (71.0) |  |  |
| Religion (*n* = 88) |  |  |  |  | |
| Yes | 40 (45.5) | 28 (49.1) | 12 (38.7) | .88 | |
| No | 48 (54.5) | 29 (50.9) | 19 (61.3) |  |  |
| Hospitalization period (days; *n* = 86) | 6.65 (12.18) | 9.00 (14.49) | 2.48 (3.74) | 3.15^**^ | |
| *Notes*. ^†^Diagnosis based on the CAM criteria; ^‡^single/divorced/widowed; ^*^ *p* < .05; ^**^ *p* < .01. | | | | |  |

|  | Sleep-wake cycle | Perceptual disturbances | Delusions | Lability of affect | Language | Thought process | Motor agitation | Motor retardation | Orientation | Attention | Short-term memory | Long-term memory | Visuospatial ability |  |
| --- | --- | --- | --- | --- | --- | --- | --- | --- | --- | --- | --- | --- | --- | --- |
| Class 1 | 0.891 | 0.364 | 0.073 | 0.291 | 0.236 | 0.436 | 0.346 | 0.364 | 0.418 | 0.745 | 0.545 | 0.127 | 0.255 |  |
| Class 2 | 2.351 | 0.473 | 0.347 | 0.354 | 1.939 | 1.758 | 0.309 | 2.342 | 2.594 | 2.409 | 2.653 | 2.004 | 2.288 |  |
| Class 3 | 2.67 | 2.255 | 1.158 | 1.481 | 1.62 | 2.055 | 2.714 | 0.38 | 2.745 | 2.526 | 2.093 | 1.143 | 2.054 |  |
| *Note*. Class 1: Low on all symptoms (*n* = 55); Class 2: High only on core symptoms (*n* = 17); Class 3: High on all symptoms (*n* = 21). | | | | | | | | | | | | | | |

Supplementary Table2. Delirium symptom levels in latent profiles
